# Antimicrobial compounds were isolated from the secondary metabolites of *Gordonia,* a resident of intestinal tract of *Periplaneta americana*

**DOI:** 10.1186/s13568-021-01272-y

**Published:** 2021-07-30

**Authors:** Yan Ma, Minhua Xu, Hancong Liu, Tiantian Yu, Ping Guo, Wenbin Liu, Xiaobao Jin

**Affiliations:** 1 School of Life Sciences and Biopharmaceutics, Guangdong Pharmaceutical University, Guangzhou, 510006 China; 2 Guangdong Provincial Key Laboratory of Pharmaceutical Bioactive Substances, Guangdong Pharmaceutical University, Guangzhou, 510006 China

**Keywords:** *Gordonia*, Secondary metabolites, Antimicrobial activity, Antiproliferative activity, *Periplaneta americana*

## Abstract

*Gordonia* sp. are members of the actinomycete family, their contribution to the environment improvement and environmental protection by their biological degradation ability, but there are few studies on the antimicrobial activity of their secondary metabolites. Our team isolated and purified an actinomycete WA 4-31 from the intestinal tract of *Periplaneta americana*, firstly identified the strain WA 4-31 by the morphological characteristics and the phylogenetic analyses, and found it was completely homologous to the strain of *Gordonia terrae* from the Indian desert. Meanwhile, actinomycin D (**1**), actinomycin X2 (**2**), mojavensin A (**3**) and cyclic (leucine-leucne) dipeptide (**4**) were obtained from the EtOAc extract from the broth of WA 4-31. Compounds **1**–**4** showed anti-fungus activities against *Candida albicans*, *Aspergillus niger*, *A. fumigatus* and *Trichophyton rubrum*, also anti-MRSA and inhibited *Escherichia coli* in different degree. Interestingly, we found when **3** was mixed with **4** with ratio of 1:1, the activity of the mixture on anti-*Candida albicans* was better than the single. Besides, compounds **1–3** had varying degrees of antiproliferative activities on CNE-2 and HepG-2 cell lines. These indicated that *Gordonia* rare actinomycete from the intestinal tract of *Periplaneta americana* possessed a potential as a source of active secondary metabolites.

**Supplementary Information:**

The online version contains supplementary material available at 10.1186/s13568-021-01272-y.

## Introduction

Microbial secondary metabolites are one of the main sources of bioactive natural products and are also one of the main sources of drugs. It is estimated that around 75% of all antibiotics are derived from secondary metabolites produced by filamentous actinomycete, especiely *Streptomyces* species. But over time, the continual rediscovery of known compounds from secondary metabolites of actinobacteria has resulted. Consequently, special extreme environment and growing conditions for bacteria and Fungi raised much concerns in recent years (Sayed et al. 2020), such as deep-sea environment, with characteristic features of high salinity, high pressure, low temperature and low nutrition (Sivalingam et al. 2019; Zain et al. 2019); cold polar regions (Durán et al. 2019); deserts (Velez et al. 2018; Riahi et al. 2019) or endogenous environment in insects and plants. (Torres-Mendoza et al. 2020; Xu et al. 2020)

Endogenous microorganisms of insects and plants have attracted much attention in recent years. They play important role of acting as reservoirs of novel bioactive secondary metabolites that serve as a potential candidate for antimicrobial, anti-insect, anticancer and many more properties (Tanvir et al. 2017). In order to cope with the emergence of drug resistance, scientific efforts have been aimed at the bioprospecting of microorganisms' secondary metabolites, with special emphasis on the search for antimicrobial natural products derived from endophytes (Kaur and Arora 2020; Mastan et al. 2020; Martinez-Klimova et al. 2017; Silva et al. 2018). Besides, in the existing research reports of insect microorganisms, most of them focus on pest control, biological agents, ecological protection and ecological diversity, and the study on antimicrobial activity of their secondary metabolites is less (Dantur et al. 2015; Guo et al. 2017; Zheng et al. 2020). Our previous study showed that the endobacteria in the intestinal tract of *Periplaneta americana* exhibited various antimicrobial activities against all tested pathogens (Fang et al. 2018; Chen et al. 2020).

Rare actinomycetes have attracted more attention with a hope to discover new antibiotics. At present, some antibiotics that have been used in clinic, such as gentamicin, erythromycin, vancomycin and rifampicin, are all from rare actinomycetes (Abidi et al. 2020). Rare actinomycete *Gordonia* are usually used for the environment improvement and environmental protection by its biological degradation ability, but there are few studies on its antimicrobial activity (Shintani et al. 2019). Our team isolated and identified a *Gordonia* strain WA 4-31 with anti-*Candida albicans* activity from the intestinal tract of *Periplaneta americana.* Meanwhile, four compounds were isolated and purified from the ethyl acetate extract of fermentation broth of the *Gordonia* strain WA 4-31 with antimicrobial activity, especially compounds1-3 Actinomycin D (**1**), Actinomycin X_2_ (**2**), Mojavensin A (**3**) showed better anti-fungal and anti-cancer activities. The study reported the antimicrobial compounds isolated from *Gordonia* from the intestinal tract of *Periplaneta americana* for the first time. These indicated that *Gordonia* rare actinomycetes possessed a potential as a resource of active secondary metabolites.

## Material and methods

### Microorganisms, cell lines

The *Gordonia terrae* Strain WA 4-31(MH613773) was isolated from the intestinal tract of *Periplaneta americana* and deposited in Guangdong Microbial Culture Collection Center (GDMCC NO.1.2521). The tested strain including four kinds of pathogenic fungi: *Candida albicans* ATCC 10231, *Trichophyton rubrum* ATCC 60836, *Aspergillus niger* ATCC 16404, *Aspergillus fumigatus* ATCC 96918, and four kinds of pathogenic bacteria: m*ethicillin-resistant Staphylococcus aureus* (MRSA) ATCC 43300, *Staphylococcus aureus* ATCC 25923, *Escherichia coli* ATCC 25922 and *Pneumobacillus* ATCC 13883, were obtained from Guangdong Institute of Microbiology. CNE-2 Human nasopharyngeal carcinoma cell line, HepG2 human hepatocellular carcinoma cells were purchased from Experimental Animal Center of Sun Yat-sen University and Shanghai Cell Biology Institutes respectively.

### Media and chemicals

Microbiological media were purchased from Guangdong Huankai Microbial Co.(Guangzhou, China). The genomic DNA isolation kit was purchased from TIANGEN Biotech (Beijing, China). Silica gel used for extraction and column chromatography were obtained from Qingdao Ocean Chemical Co. (Qingdao, China). Methanol and Acetonitrile for High-performance liquid chromatography (HPLC) were purchased from ThermoFisher (Thermo Fisher Scientific, United States). Other solvents were obtained from Guangdong Guanghua Sci-Tech Co.(Guangzhou, China).

### Identification of strain WA 4-31

The *Gordonia* strain WA 4–3 was inoculated on Gauze’s No. 1 medium and incubate at 28 °C for 3 days. Three days later, the single colony was selected and cultivated in the ISP-1 seed medium at 28 °C for 2 days. The morphology and surface characteristics of the bacterial colonies were examined using a scanning electron microscope (Hitachi S-3400 N, Japan). Whole genomic DNA of the strain was extracted according to the manufacturer’s instructions of the genomic DNA isolation kit. The 16S rDNA gene was then amplifified by PCR, using universal bacterial primer (27F: 5’-AGA GTT TGA TCC TGG CTC AG-3’ and 1492R: 5’-TAC GGC TAC CTT GTT ACGACT T-3’). PCR products were visualized by electrophoresis and was sequenced in Invitrogen (Guangzhou, China). The resulting sequence was compared to all sequences available in GenBank using the BLAST software from the National Center for Biotechnology Information (NCBI) website (http://www.ncbi.nlm.nih.gov/). The phylogenetic tree was constructed by MEGA.

### Formation, extract, isolation, and purification of WA 4-31

The ISP 2 medium was used as the production culture. A 9 mL portion of the seed culture was transferred into a 500-mL Erlenmeyer flask containing 300 mL of the ISP 2 medium and incubated on rotary shakers (160 rpm) at 28 °C for 21 days. The total 63L of culture broth was extracted 3 times with an equivalent volume of ethyl acetate. After extraction and evaporation, 13.4 g crude extract was obtained, which was isolated to given 20 fractions (1–20) using silica gel column with mobile phase dichloromethane:methanol (100:0–0:100, V/V). The crude extract and every fraction were tested for antimicrobial activity against Candida albicans ATCC 10231 by Oxford Cup method (Tao et al. 2016). Finally, the active fractions were further purified for separation of compounds by Reverse-phase high performance liquid chromatography (RP-HPLC). RP-HPLC was performed on a semi-prepared HPLC system(Waters e2535-2489, USA) with YMC-Pack ODS-AQ C18 column (250 × 10.0 mm, YMC AQ12S05-2546WT, Japan) using a UV–VIS detector and a 200μL injection loop at 25 ℃. Deionized water and methanol were used as the mobile phases. The flow rate was set to 1.0 mL/min, and UV detection was recorded at λ = 280 nm. Chromatography was performed using a linear gradient of 5–90% methanol over 65 min, and then held at 90% methanol for 10 min.

### Spectroscopic analysis

The molecular weight of the purified compounds was determined using mass spectrometry and their structure was characterized by 1H NMR and 13C NMR. The compounds were fully dissolved in methanol with a concentration of about 1 mg/L, filtered with a 0.22 μm filter membrane, and then mass spectra was obtained within the range of m/z100-1500 by Triple-quadrupole mass spectrometer (Thermo Scientific TSQ Endura™, USA). Selecting appropriate 600μL of deuterated reagent to dissolve 10.0 mg of the compounds, the proton and carbon nuclear magnetic resonance (NMR) spectra were recorded at 600 MHz using a spectrometer (Brucker AVANCE III 600 M, Germany).

### Determination of minimum inhibitory concentration (MIC)

Minimum inhibitory concentration (MIC) measurements of compounds against the tested strain were performed by a microdilution method. The compounds were dissolved in DMSO and then diluted by the twofold dilution method. 100 µL bacterial suspension or fungi suspension (10^6^ CFU/mL) were added to the 96-well plates. Then 100 µL compounds were added to the 96-well plates respectively. The final concentrations of each sample in the wells were 512, 256, 128, 64, 32, 16, 8, 4, 2, 1, 0.5 and 0.25 µg/mL. Amphotericin B(Aladdin A105482, China), Vancomycin (Tianfu Chemical 1404-93-9, China), Ampicillin (Tianfu Chemical 7177-48-2, China) and Ciprofloxacin (Macklin C824343, China) were used as positive drugs for fungi, MRSA, *Pneumococcus* and *Escherichia coli* respectively. The MIC value of the compounds was determined as the lowest concentration that completely inhibited bacterial growth after 48 h with incubation at 37℃, while the fungi was incubated at 28℃. All experiments were performed in triplicate.

### Filter paper diffusion method

Antifungal activity against *Candida albicans* was tested using the filter paper diffusion method. Sterile filter paper discs (6 mm) were taken, soaked sufficiently in different samples for 5 min and set aside. Fungi suspension was dipped with sterile cotton, and uniformly applied in the culture media. Fully soaked filter paper was placed in the *C. albicans* containing plates and cultured for 24 h at 28 °C. Amphotericin B (32 μg/mL) was used as positive control. The experiment was performed in triplicate and the mean of the diameter of the inhibition zones was calculated.

### MTT assay

3-(4,5-dimethylthiazol-2-yl)-2,5-diphenyl tetrazolium bromide (MTT, Sigma M2128, USA) assay was carried to evaluate cell viability. 5 × 10^3^ cells suspended in 100 μl DMEM medium were added into a 96-well plate and treated with different concentrations of compounds for 24 h /48 h /72 h. After then, 10 μL of 0.5 mg/mL MTT reagent was added to each well for an additional 4 h. The purple formazan crystals were dissolved with 100 μL DMSO per well for further quantitative measurement at an absorbance of 570 nm using a microplate spectrophotometer (BioTek Elx808, USA). Experiments were independently repeated three times.

## Results

### Identification of the strain WA 4-31

**Fig. 1:**
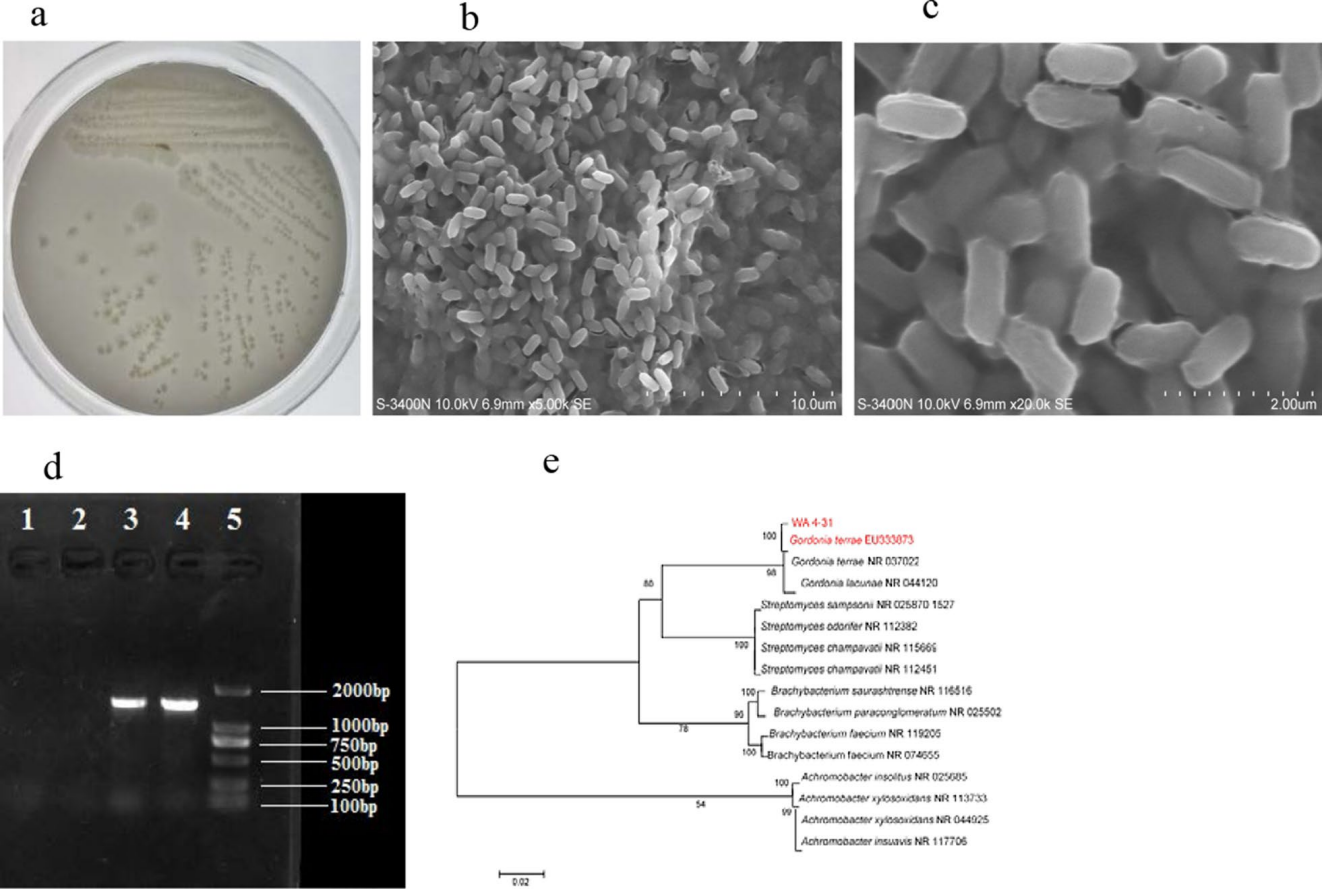
Results of strain identification **a** Colonies of Strain WA4-31 on Gauze’s medium. **b** Observation Results of Strain WA4-31 under Scanning Electron Microscope of 5000 × . **c** Observation Results of Strain WA4-31 under Scanning Electron Microscope of 20,000 × . **d** Detection of PCR Products of 16S rDNA by Agarose Gel Electrophoresis. 1、2: Black control; 3、4:WA4-31 16S rDNA; 5:2000 DNA marker. **e** Phylogenetic Tree of Strain WA 4-31. Maximum-likelihood phylogenetic tree based on 16S rDNA gene sequences showing the positions of strain WA 4-31, the type strains of other *Gordonia* and representatives of some other related taxa. Only bootstrap values (expressed as percentages of 1,000 replications) greater than 50% are shown at branching points. Bar, 0.02 substitutions per nucleotide position

The colonies of strain WA 4-31 on Gauze’s medium were milky yellow. The surface of the colony was smooth and moist, and its edge was irregularly convex (Fig. 1A). The strain WA 4-31 was observed to be short rod-shaped and unbranched under electron microscope (Fig. 1B, C). The 16S rRNA sequence of this strain WA 4-31 was submitted to GenBank of NCBI, which was compared by BLAST and homologous to *Gordonia terrae* from a cold desert of the Indian (EU333873) by 100% (Fig. 1E). The accession number was MH613773, and the deposit number was GDMCC NO.1.2521.

### Isolation and purification of secondary metabolites

**Fig. 2:**
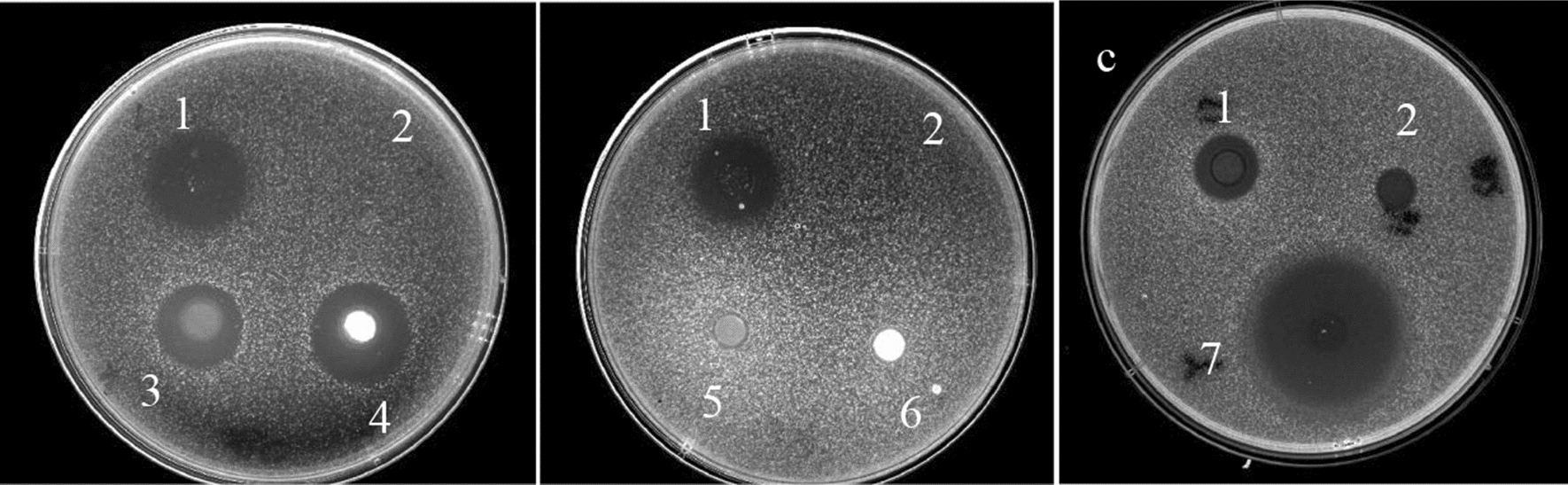
The antibacterial activity of different fractions against *Candida albicans* with Oxford Cup method **a** The 3^rd^ and the 4th fractions had anti-*Candida albicans* activity, **b** while the 5th and the 6th did not, **c** The 10th fraction had stronger antibacterial activity. 1: Positive drug: Amphotericin B, 32 μg/mL; 2: Blank control: methanol solution; 3–7: Methanol solutions with different fractions, 5 mg/mL

The crude ethyl acetate extract (about 13.4 g) was firstly separated by silica gel column chromatography eluting with a gradient system of CH_2_Cl_2_: CH_3_OH to yield 20 fractions. Six fractions showed activity against *Candida albicans* ATCC 10231 by Oxford Cup method, especially the tenth fraction was strongest, the diameters of inhibition zones of which was 36.0 ± 2.0 mm, while the others did not show activity (Fig. 2; Table 1). These fractions with anti-*Candida albicans* activity were purified by RP-HPLC to offer compound 1 (7.7 mg), compound 2 (24.2 mg), compound 3 (34.1 mg) and compound 4 (35.9 mg).

**Table 1:** The Inhibition Zone size of the fractions for *Candida albicans* with Oxford cups method

	3rd Fraction	4th Fraction	10th Fraction	11th Fraction	15th Fraction	16th Fraction	Positive drug
	Diameter of the inhibition zone (mm) ( n = 3, means ± SD)						
*Candida albicans* ATCC 10,231	13.0 ± 1.5	10.5 ± 1.0	36.0 ± 2.0	16.0 ± 1.0	13.5 ± 1.0	12.0 ± 1.5	15.0 ± 1.0

### Identification of compounds

**Fig. 3:**
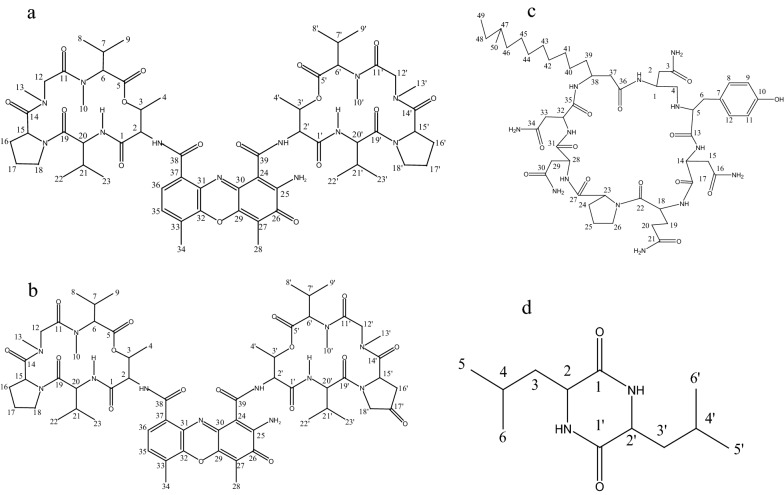
Molecular Structure of compound 1–4 **a** compound 1: Actinomycin D. **b** compound 2: Actinomycin X_2._ **c** Compound 3: Mojavensin A. **d** compound 4: cyclic (leucine-leucine) dipeptide

Compound 1, orange powder; HR-ESI–MS m/z 1255.46 [M + H] + , m/z 1277.39 [M + Na] + . Compound 2, orange powder; HR-ESI–MS m/z 1269.42[M + H] + , m/z 1291.44 [M + Na] + . Compound 3, white solid; HR-ESI–MS m/z 1086.51[2 M + H] + , m/z 1107.43 [M + Na] + . Compound 4, colorless needle-like solid; HR-ESI–MS m/z 227.15 [M + H] + . The chemical structure of compounds 1–4 was identified based on the spectral information relating to ^1^H NMR and ^13^C NMR spectral data (See Additional files 1 and 2), which were respectively confirmed to be Actinomycin D (**1**), Actinomycin X_2_ (**2**), Mojavensin A (**3**) and cyclic (leucine-leucine) dipeptide (**4**) by comparing the microspectrum, SciFinder and related references (Fig. 3).

### Antimicrobial activity of compounds

The MIC experiment found that compounds 1–4 have broad spectrum antimicrobial activity (Table 2), especially compound 3 Mojavensin A had the best effect on the four kinds of the tested fungi: *Candida albicans*, *Trichophyton rubrum*, *Aspergillus fumigatus* and *Aspergillus niger*. Compound 4 (leucine) -Leucine) dipeptide has the strongest inhibitory effect on *E. coli* at 32 μg/mL. In addition, compounds 1 Actinomycin D and compounds 2 Actinomycin X2 have the best effects against MRSA (ATCC 43,300), with an inhibitory effect of 0.25 μg/mL.

**Table 2:** The Minimal inhibitory concentration (MIC) of Compounds against the tested strains (μg/mL)

	Compound 1 Actinomycin D	Compound 2 Actinomycin X2	Compound 3 Mojavensin A	Compound 4 cyclic (leucine-leucine) dipeptide
*Candida albicans* ATCC 10231	32	128	64	> 256
*Trichophyton rubrum* ATCC 60836	128	128	16	> 256
*Aspergillus niger* ATCC 16404	128	128	32	128
*Aspergillus fumigatus* ATCC 96918	128	128	64	> 256
MRSA ATCC43300	0.25	0.25	> 256	> 256
*S.aureus* ATCC25923	64	128	> 256	> 256
*E.coli* ATCC 25922	64	128	32	32
*K.peneumoniae* ATCC 13883	64	128	> 256	> 256

**Fig. 4:**
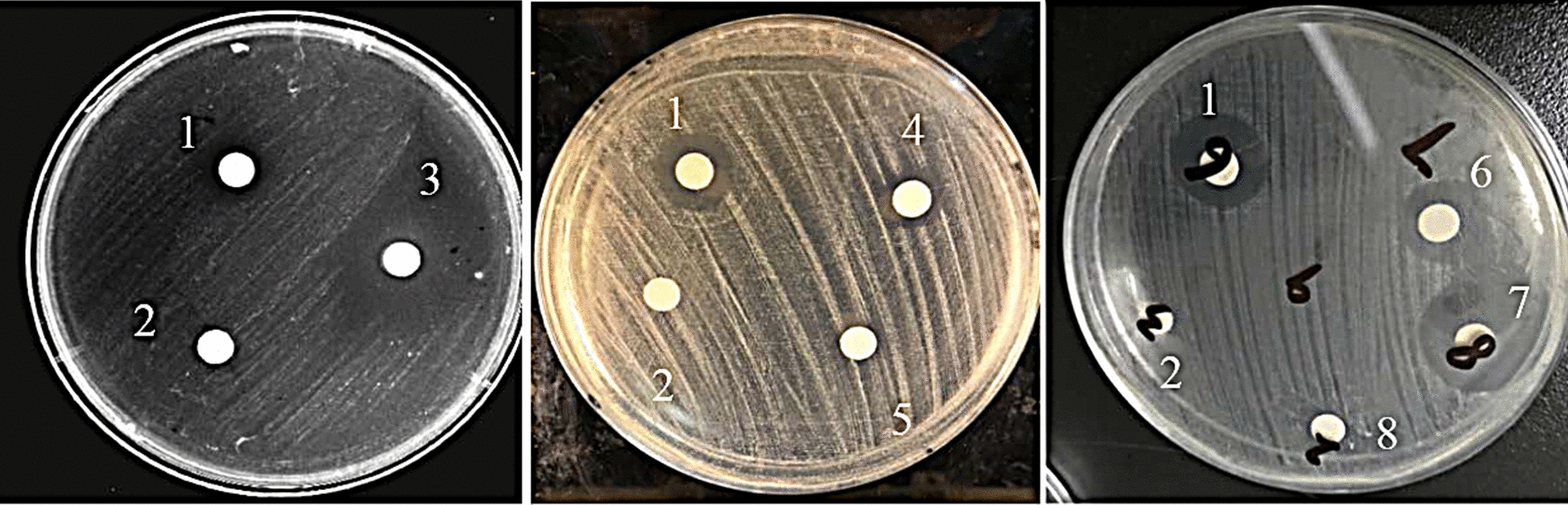
Activity of various samples against *Candida albicans* by Filter paper method 1: Positive drug: Amphotericin B, 32 μg/mL; 2: Blank control: methanol solution; 3:the tenth fraction dissolved in methanol, 1 mg/mL; 4: Mojavensin A dipeptide dissolved in methanol, 512 μg/mL; 5: Cyclic (leucine-leucine) dipeptide dissolved in methanol, 512 μg/mL; 6: Mojavensin A: cyclic (leucine-leucine) dipeptide = 2: 1; 7: Mojavensin A: cyclic (leucine-leucine) dipeptide = 1: 1; 8: Mojavensin A: cyclic (leucine-leucine) dipeptide = 1: 2

Interestingly, Mojavensin A and cyclic (leucine-leucine) dipeptide, which were separated from the tenth fraction with the strong activity against *Candida albicans* (Fig. 4), mixed with different proportions had stronger activity against *Candida albicans* than single. When the ratio was 1:1, the activity against *Candida albicans* was the strongest, but it still did not reach the strength of the tenth fraction (Table 3).

**Table 3:** The Inhibition Zone size of various samples against *Candida albicans* by Filter paper method

	Compound 3 Mojavensin A	Compound 4 cyclic (leucine-leucine) dipeptide	Mojavensin A: cyclic (leucine- leucine) dipeptide = 1: 1	Mojavensin A: cyclic (leucine- leucine) dipeptide = 2: 1	Mojavensin A: cyclic (leucine- leucine) dipeptide = 1: 2	The tenth fraction	Positive drug
	Diameter of the inhibition zone (mm) ( n = 3, means ± SD)						
*Candida albicans* ATCC 10,231	8.5 ± 0.5	-	14.5 ± 1.0	11.5 ± 1.5	8.0 ± 1.5	19.0 ± 1.0	11.5 ± 0.5

### Antiproliferative activity of compounds

**Fig. 5:**
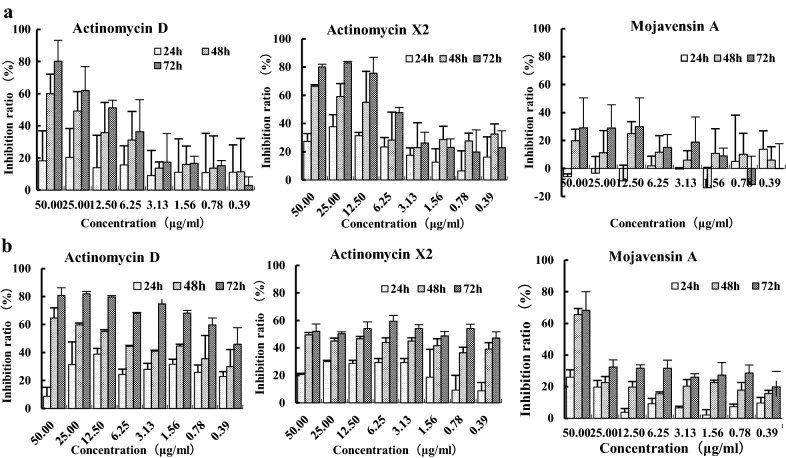
The cytotoxicity (MTT ASSAY) results of compounds on HepG-2cells (**a**) and CNE-2 cells (**b**) at the different concentrations for 24–72 h

Cytotoxicity of Compounds 1–3 Actinomycin D, Actinomycin X_2_ and Mojavensin A for HepG-2 and CNE-2 cells was performed by MTT at different time. Results show that compounds 1–3 could inhibited cell proliferation in a time-dependent manner (Fig. 5). Meanwhile the inhibitory effect of them for HepG-2 had the concentration correlation. The antiproliferative effect of Actinomycin D and Actinomycin X_2_ was more effectively than Mojavensin A at the Concentration of less than 50 μg/ml. According to the inhibition rate of different concentrations, the half-maximal inhibitory concentration (IC_50_) of the compounds on HepG-2 and CNE-2 cells were calculated using the probability distribution of SPSS13.0 (Table 4).

**Table 4:** The half-maximal inhibitory concentration (IC_50_) of compounds on HepG-2 and CNE-2 cells for 24–72 h (μg/mL)

	HepG-2			CNE-2		
	24 h	48 h	72 h	24 h	48 h	72 h
Actinomycin D	> 200	29.74	12.16	> 200	15.02	1.24
Actinomycin X_2_	> 200	14.68	5.37	> 200	84.38	0.93
Mojavensin A	-	> 200	> 200	> 200	130.72	51.03

## Discussion

The growing resistance of microorganisms towards antibiotics has become a serious problem around the world. Therapeutics with novel chemical scaffolds and/or mechanisms of action are urgently needed to combat infections caused by multidrug resistant pathogens, including bacteria, fungi and viruses. Development of novel antimicrobial agents still highly depends on the new natural products. At present, most antimicrobial drugs used in medicine are of natural origin. Among the natural producers of bioactive substances, actinobacteria continue to be an important source of novel secondary metabolites for drug application (Jakubiec-Krzesniak et al. 2018). It was estimated that from the first report of streptothricin in 1942 and streptomycin a year later, the order Actinomycetales had yielded about 3000 known antibiotics (Clardy et al. 2006). However, with the known antibiotics are discovered repeatedly, there are fewer new antibiotics found from *Streptomyces*. Rare actinomycetes have been paid more attention as the hope of discovering new antibiotics. Ramesh et al. reported that 167 new bioactive compounds produced by 58 different rare actinomycete species representing 24 genera. A total of 97 new species, representing 9 novel genera and belonging to 27 families of marine rare actinomycetes had been reported from mid-2013 to 2017 (Subramani and Sipkema 2019). About 20 years ago, rare actinomycete *Gordonia* was described as a genus, and there are 39 different species have been identified so far (Sowani et al. 2018). Some *Gordonia* species cause a broad spectrum of diseases in healthy and immunocompromised individuals. Besides, these bacteria can produce useful secondary metabolites that may be used in various industries (Nahurira et al. 2019).

In this study, we validated the endogenous *Gordonia* isolated from the intestinal tract of *Periplaneta americana* by sequence and phylogenetic tree construction and found that the strain was 100% homologous to *Gordonia terrae* from the Indian desert. Colony conformed to the biomarker of *Gordonia* by colony phenotype. We named this *Gordonia* strain WA 4-31. Previous studies have shown that the endogenous environment of insects is unique and the natural products form the endogenous environment of insects may be different from the common environment in the land. Therefore, it is speculated that insect endobacteria may produce precious and interesting natural compounds (Beemelmanns et al. 2016; Newman and Cragg 2016; Chevrette et al. 2019). Insects are a highly diverse group, exploit a wide range of habitats, and harbor bacterial symbionts of largely unknown diversity. Insect-associated bacterial symbionts are underexplored but promising sources of bioactive compounds (Martinez et al. 2019). Lee et al. (2018) reported the discovery of three new cyclic tripeptides: natalenamides A-C. These compounds were identified from the culture broth of the fungus-growing termite-associated *Actinomadura* sp. RB99. Li et al. (2020) reported two new compounds, versicolones A and B and three known pyrone derivatives were isolated from the insect-associated fungus *Aspergillus versicolor*. At the present study, four antimicrobial compounds were isolated from the secondary metabolite of the strain WA4-31. Although these compounds, Actinomycin D, Actinomycin X2 and Mojavensin A were reported, which found in *Gordonia* is the first time. There was no report that they were found in secondary metabolites at the same time so far.

In our study, Actinomycin D and Actinomycin X_2_ showed extremely strong activity on MRSA, reached 0.25 μg/mL respectively, which was consistent with the existing reports (Rathod et al. 2018; Sharma and Manhas 2019). These two Actinomycins showed general resistance to the remaining tested pathogens, which was different, compared with some reports. Xiong et al. found that Actinomycin X_2_ had strong antibacterial activity against several Gram-positive and Gram-negative bacteria examined (*Staphylococcus aureus*, *Pseudomonas solanacearum*, *Escherichia coli*, etc.) and was especially effective against *S. aureus,* with MIC50, 0.002 μg/ml; MIC90, 0.017 μg/ml (Xiong et al. 2012). Actinomycin D from the study by Zhang et al. were evaluated for their activity against the growth of methicillin-resistant *S. aureus*, *E. coli*, and *C. albicans* using the micro broth dilution method and the results showed that Actinomycin D significantly inhibited the growth of both bacteria and fungi with MIC values of 0.08 to 9.96 μM (Zhang et al. 2016). Among compounds, Mojavensin A had the best inhibitory activity against *Aspergillus niger*, *Trichophyton rubrum* and *Aspergillus fumigatus*, reaching 32, 16 and 64 μg/mL. The inhibitory effect of compound 3 Mojavensin A on *Escherichia coli* was 32 μg/mL. Mojavensin A was found in 2012 for the first time (Ma et al. 2012). In another report said mojavensins displayed moderate antagonism and dose-dependent activity against several formae speciales of *Fusarium oxysporum* and presented surface tension activities. These properties demonstrated that these lipopeptides may be useful as biological control agent to fungal plant pathogens (Ma and Hu 2014). These reports provided good evidence for our study on antimicrobial activity in this article.

In addition, we found a more interesting phenomenon. In the process of separating and purifying the secondary metabolites of strain WA 4-31, it was found that one of the fractions showed strong activity against *Candida albicans* (Fig. 2; Table 1). Compound 3 Mojavensin A and compound 4 cyclic (leucine-leucine) dipeptide were obtained by further separation and purification from this fraction. The anti-*Candida albicans* activity of these two compounds was determined, and the activity of single compound was not outstanding. Then we mixed these two compounds according to different proportions, and it was found that the activity was obviously improved, but it still did not reach the strength of the fraction (Fig. 4; Table 3). Antimicrobial resistance threatens a resurgence of life-threatening bacterial infections and the potential demise of many aspects of modern medicine. In order to delay the problem of antimicrobial resistance, people have extensively studied the synergistic effect of drugs in recent years. Combinations of antibiotics and of antibiotics with non-antibiotic activity-enhancing compounds offer a productive strategy to address the widespread emergence of antibiotic-resistant strains (Tyers and Wright 2019; Zheng et al. 2018). As there are still other complex bioactive components in the fractions that have not been separated, the synergistic effect and its mechanism among the specific compounds with strong anti-*Candida albicans* activity have not yet been determined, which needs further study. The inhibitory activities of all compounds on different bacteria and fungi were not consistent, so it was speculated that their coexistence was complementary for the host. Perhaps the host used them to fight against different pathogenic microorganisms to adapt to various living environments. At the same time, it also showed that the diversity and richness of secondary metabolites were conducive to the adaptation of microorganisms to their living environment for microorganisms themselves, which is of great significance (Cheng et al. 2020).

Through our work, we identified a rare actinomycete strain WA 4-31 from the intestinal tract of *Periplaneta americana* as *Gordonia*. Four compounds with antimicrobial activity, Actinomycin D, Actinomycin X_2_, Mojavensin A and cyclic (leucine-leucine) dipeptide were isolated and purified from the secondary metabolites of the strain WA 4-31. We determined their antimicrobial activity and inhibitory effect on the growth of CNE-2 cells and HepG-2 cells. In addition, we found that Mojavensin A and cyclic (leucine-leucine) dipeptide had a synergistic effect after being mixed in different proportions, which could enhance the anti-*Candida albicans* activity. This phenomenon was a good enlightenment for us, maybe we can continue our in-depth study in the follow-up study on antimicrobial activity.

## Supplementary Information

**Additional file 1**: **Fig. S1**. Identification of compound 1: Actinomycin D. (a) Mass spectrometry. (b) Proton (1H) nuclear magnetic resonance. (c) Carbon (13C) nuclear magnetic resonance spectrum of the compound 1 in Chloroform-d. **Fig. S2**. Identification of compound 2: Actinomycin X2. (a) Mass spectrometry. (b) Proton (1H) nuclear magnetic resonance. (c) Carbon (13C) nuclear magnetic resonance spectrum of the compound 2 in Chloroform-d. **Fig. S3**. Identification of compound 3: Mojavensin A. (a) Mass spectrometry. (b) Proton (1H) nuclear magnetic resonance. (c) Carbon (13C) nuclear magnetic resonance spectrum of the compound 3 in DMOS-6d. **Fig. S4**. Identification of compound 4: cyclic (leucine-leucine) dipeptide. (a) Mass spectrometry. (b) Proton (1H) nuclear magnetic resonance. (c) Carbon (13C) nuclear magnetic resonance spectrum of the compound 4 in Chloroform-d.

**Additional file 2**: **Table S1**. NMR data correlations of Actinomycin D (Compound 1). **Table S2**. NMR data correlations of Actinomycin X2 (Compound 2). **Table S3**. NMR data correlations of Mojavensin A (Compound 3). **Table S4**. NMR data correlations of cyclic (leucine-leucine) dipeptide (Compound 4).

## Data Availability

All the data analysed in this study are included in this article.
