# Supplementary material for: Antimicrobial compounds were isolated from the secondary metabolites of Gordonia, a resident of intestinal tract of Periplaneta americana
Source: AMB Express. 2021 Jul 30;11:111. doi: 10.1186/s13568-021-01272-y (PMC8324697; doi:10.1186/s13568-021-01272-y)
Supplement: Supplementary file 1 — Additional file 1: Fig. S1. Identification of compound 1: Actinomycin D. (a) Mass spectrometry. (b) Proton (1H) nuclear magnetic resonance. (c) Carbon (13C) nuclear magnetic resonance spectrum of the compound 1 in Chloroform-d. Fig. S2. Identification of compound 2: Actinomycin X2. (a) Mass spectrometry. (b) Proton (1H) nuclear magnetic resonance. (c) Carbon (13C) nuclear magnetic resonance spectrum of the compound 2 in Chloroform-d. Fig. S3. Identification of compound 3: Mojavensin A. (a) Mass spectrometry. (b) Proton (1H) nuclear magnetic resonance. (c) Carbon (13C) nuclear magnetic resonance spectrum of the compound 3 in DMOS-6d. Fig. S4. Identification of compound 4: cyclic (leucine-leucine) dipeptide. (a) Mass spectrometry. (b) Proton (1H) nuclear magnetic resonance. (c) Carbon (13C) nuclear magnetic resonance spectrum of the compound 4 in Chloroform-d. [file 13568_2021_1272_MOESM1_ESM.docx]

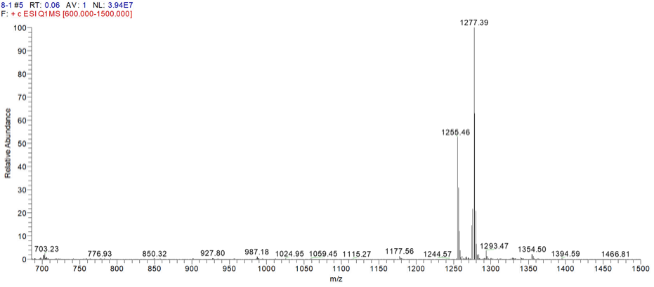


a

b

c

**Fig. 1 Identification of compound 1: Actinomycin D**

(a) Mass spectrometry. (b) Proton (1H) nuclear magnetic resonance. (c) Carbon (13C) nuclear magnetic resonance spectrum of the compound 1 in Chloroform-d.

a


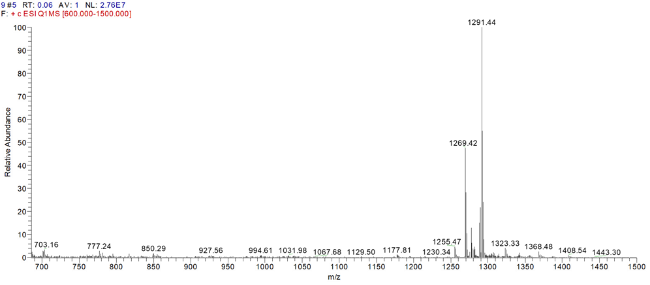


c

b

**Fig. 2 Identification of compound 2: Actinomycin X_2_**

(a) Mass spectrometry. (b) Proton (1H) nuclear magnetic resonance. (c) Carbon (13C) nuclear magnetic resonance spectrum of the compound 2 in Chloroform-d.


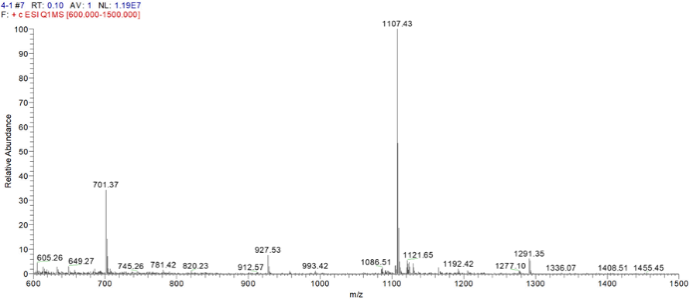


c

b

a

**Fig. 3 Identification of compound 3: Mojavensin A**

(a) Mass spectrometry. (b) Proton (1H) nuclear magnetic resonance. (c) Carbon (13C) nuclear magnetic resonance spectrum of the compound 3 in DMOS-6d.


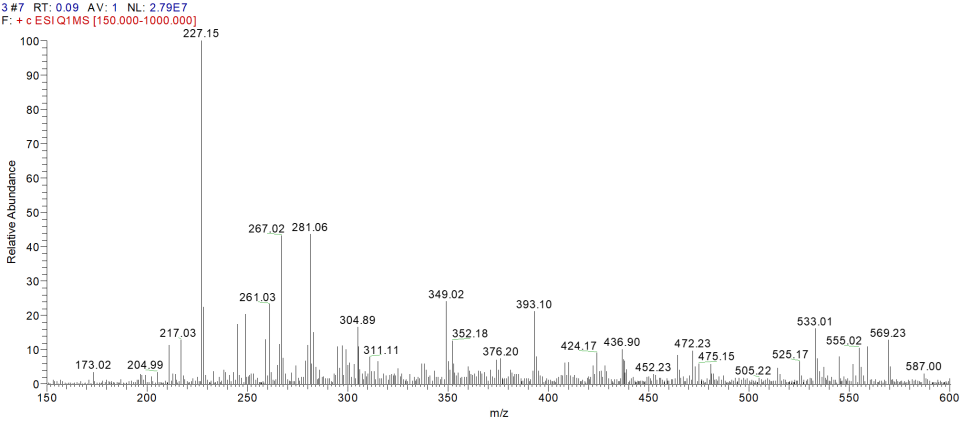


a

b

c

**Fig. 4 Identification of compound 4: cyclic (leucine-leucine) dipeptide**

(a) Mass spectrometry. (b) Proton (1H) nuclear magnetic resonance. (c) Carbon (13C) nuclear magnetic resonance spectrum of the compound 4 in Chloroform-d.
