# Supplementary material for: Antimicrobial compounds were isolated from the secondary metabolites of Gordonia, a resident of intestinal tract of Periplaneta americana
Source: AMB Express. 2021 Jul 30;11:111. doi: 10.1186/s13568-021-01272-y (PMC8324697; doi:10.1186/s13568-021-01272-y)
Supplement: Supplementary file 2 — Additional file 2: Table S1. NMR data correlations of Actinomycin D (Compound 1). Table S2. NMR data correlations of Actinomycin X2 (Compound 2). Table S3. NMR data correlations of Mojavensin A (Compound 3). Table S4. NMR data correlations of cyclic (leucine-leucine) dipeptide (Compound 4). [file 13568_2021_1272_MOESM2_ESM.docx]

**Table 1 NMR data correlations of Actinomycin D (Compound 1)**

| **Position** | **13C NMR** | **H NMR** | **Position** | **13C NMR** | **H NMR** |
| --- | --- | --- | --- | --- | --- |
| **1** | 168.6 |  | **16’** | 31.3 |  |
| **1’** | 167.7 |  | **17** | 23.1 |  |
| **2** | 55.2 | 4.61(1H, d) | **17’** | 22.9 |  |
| **2’** | 54.9 | 4.50(1H, d) | **18** | 47.6 |  |
| **3** | 75 | 5.16(1H, dd) | **18’** | 47.4 |  |
| **3’** | 75.1 | 4.78(1H, dd) | **19** | 173.4 |  |
| **4** | 17.8 | 1.23(3H, d) | **19’** | 173.4 |  |
| **4’** | 17.4 |  | **20** | 58.9 |  |
| **5** | 167.7 |  | **20’** | 58.2 |  |
| **5’** | 166.6 |  | **21** | 31.8 |  |
| **6** | 71.4 |  | **21’** | 31.6 |  |
| **6’** | 71.2 |  | **22** | 19.2 | 1.10(3H, m) |
| **7** | 27 |  | **22’** | 19.1 |  |
| **7’** | 27 |  | **23** | 19.1 | 0.87(6H, dd) |
| **8** | 21.7 | 0.94(3H, m) | **23’** | 19 |  |
| **8’** | 21.6 |  | **24** | 101.7 |  |
| **9** | 19.3 | 0.73(6H, d) | **25** | 147.6 |  |
| **9’** | 19.3 |  | **26** | 179.1 |  |
| **10** | 39.3 | 2.86(3H, s) | **27** | 113.6 |  |
| **10’** | 39.2 | 2.65(3H, m) | **28** | 7.8 | 2.22(3H, s) |
| **11** | 166.6 |  | **29** | 145.1 |  |
| **11’** | 166.4 |  | **30** | 145.9 |  |
| **12** | 51.4 | 3.62(2H, m) | **31** | 129.1 |  |
| **12’** | 51.4 |  | **32** | 140.5 |  |
| **13** | 34.9 | 2.89(3H, s) | **33** | 127.8 |  |
| **13’** | 35 |  | **34** | 15.1 | 2.53(3H, s) |
| **14** | 173.3 |  | **35** | 130.4 | 7.13(1H, d) |
| **14’** | 173.3 |  | **36** | 125.8 | 7.61(1H, d) |
| **15** | 56.3 | 2.92(3H, s) | **37** | 132.6 |  |
| **15’** | 56.5 |  | **38** | 166.5 |  |
| **16** | 31 |  | **39** | 169.1 |  |

**Table 2 NMR data correlations of Actinomycin X_2_ (Compound 2)**

| **Position** | **13C NMR** | **H NMR** | **Position** | **13C NMR** | **H NMR** |
| --- | --- | --- | --- | --- | --- |
| **1** | 168.8 |  | **16’** | 42 |  |
| **1’** | 167.6 |  | **17** | 23.1 |  |
| **2** | 55.1 | 4.50(1H, dd) | **17’** | 208.9 |  |
| **2’** | 54.4 | 4.56(1H, dd) | **18** | 47.5 |  |
| **3** | 74.8 | 5.94(1H, d) | **18’** | 51.4 |  |
| **3’** | 74.7 | 5.20(1H, qd) | **19** | 173.6 |  |
| **4** | 17.8 | 1.25(3H, d) | **19’** | 172.8 |  |
| **4’** | 17.3 | 1.26(3H, s) | **20** | 58.6 |  |
| **5** | 167.6 |  | **20’** | 57.3 |  |
| **5’** | 166.4 |  | **21** | 32 |  |
| **6** | 71.5 |  | **21’** | 31.8 |  |
| **6’** | 71.4 |  | **22** | 19.2 | 1.13(6H, m) |
| **7** | 27 |  | **22’** | 19.2 |  |
| **7’** | 27.1 |  | **23** | 19 | 0.90(6H, t) |
| **8** | 21.8 | 0.95(3H, d) | **23’** | 18.9 |  |
| **8’** | 21.7 | 0.98(3H, d) | **24** | 101.8 |  |
| **9** | 19.3 | 0.74(6H, t) | **25** | 147.5 |  |
| **9’** | 19.3 |  | **26** | 179.1 |  |
| **10** | 39.5 | 2.89(3H, d) | **27** | 113.7 |  |
| **10’** | 39.3 |  | **28** | 7.9 | 2.24(3H, s) |
| **11** | 166.2 | 2.55(3H, s) | **29** | 145.1 |  |
| **11’** | 166 |  | **30** | 146 |  |
| **12** | 51.4 | 3.62(2H, s) | **31** | 129.3 |  |
| **12’** | 53 | 3.65(2H, s) | **32** | 140.6 |  |
| **13** | 35 | 2.93(3H, d) | **33** | 128 |  |
| **13’** | 34.9 |  | **34** | 15.2 |  |
| **14** | 174.1 |  | **35** | 130.5 | 7.36(1H, d) |
| **14’** | 173.2 |  | **36** | 126.3 | 7.60(1H, d) |
| **15** | 56.5 | 2.65(3H, s) | **37** | 132.2 |  |
| **15’** | 54.4 | 2.70(3H, s) | **38** | 166.7 |  |
| **16** | 31.1 |  | **39** | 169.1 |  |

**Table 3 NMR data correlations of Mojavensin A (Compound 3)**

| **Position** | | **13C NMR** | | **H NMR** | **Position** | **13C NMR** | | **H NMR** |
| --- | --- | --- | --- | --- | --- | --- | --- | --- |
| **1** | 50.6 | | 4.20(1H, m) | | **26** | | 47.3 | 3.33(2H, brs) |
| **2** | 36.5 | | 2.14m /2.34m(2H) | | **27** | | 172.7 |  |
| **3** | 171.3 | |  | | **28** | | 50.2 | 4.03(1H, m) |
| **4** | 173.4 | |  | | **29** | | 35.3 | 2.18m/2.56d(2H) |
| **1-NH** |  |  | 7.11(1H, s) | | **30** | | 171.5 |  |
| **3-NH_2_** |  |  | 7.88(2H, d) | | **31** | | 170.1 |  |
| **5** | 56.6 | | 3.87(3H, m) | | **28-NH** | |  | 8.37dd(1H) |
| **6** | 35.2 | | 2.34m /2.76dd(2H) | | **30-NH_2_** | |  | 6.68s/8.11s(2H) |
| **7** | 127.9 | |  | | **32** | | 50.2 | 4.44(1H, m) |
| **8,12** | 129.9 | | 6.74(1H, d) | | **33** | | 37.4 | 2.10m/2.59d(2H) |
| **9,11** | 115.2 | | 6.49(3H, d) | | **34** | | 170.4 |  |
| **10** | 155.9 | |  |  | **35** | | 170.8 |  |
| **13** | 171.5 | |  | | **32-NH** | |  | 7.66d(1H) |
| **5-NH** |  | | 7.84(1H, d) | | **34-NH_2_** | |  | 6.46s/6.80s(2H) |
| **14** | 50.8 | | 4.25(1H, m) | | **36** | | 171.5 |  |
| **15** | 36.1 | | 2.21m /2.48dd(2H) | | **37** | | 41.5 | 2.14(2H, m) |
| **16** | 171.5 | |  | | **38** | | 46.5 | 3.64(1H, m) |
| **17** | 171.5 | |  | | **39** | | 34.5 | 1.06brs(2H) |
| **14-NH** |  | | 8.36(1H, d) | | **40** | | 25.7 | 0.93m/1.12m/1.21m/1.27m/1.71m  (14H) |
| **16-NH_2_** |  | | 6.73s/6.95s(2H) | | **41** | | 28.7 |  |
| **18** | 48.7 | | 4.44(1H, m) | | **42** | | 29 |  |
| **19** | 26.6 | | 1.82m/1.86m(2H) | | **43** | | 29 |  |
| **20** | 30.8 | | 2.06(2H, m) | | **44** | | 29.2 |  |
| **21** | 174.4 | |  | | **45** | | 26.9 |  |
| **22** | 171.3 | |  | | **46** | | 36.3 |  |
| **18-NH** |  | | 6.98(1H, d) | | **47** | | 33.8 | 1.06brs(1H) |
| **21-NH_2_** |  | | 6.78s/7.11s(2H) | | **48** | | 29.2 | 0.96m/1.32m(2H) |
| **23** | 60.5 | | 4.12(1H, t) | | **49** | | 11.3 | 0.68(3H, m) |
| **24** | 29.4 | | 1.61m/2.04m(2H) | | **50** | | 19.2 | 0.90(3H, m) |
| **25** | 24.9 | | 1.71m/1.96m(2H) | | **38-NH** | |  | 6.85(1H, d) |

**Table 4 NMR data correlations of cyclic (leucine-leucine) dipeptide (Compound 4)**

| Position | 13C NMR | H NMR |
| --- | --- | --- |
| **1,1’** | 169.5 | 7.47(1H, brs) |
| **2,2’** | 53.4 | 3.95(1H, m) |
| **3,3’** | 43.9 | 1.59(2H, m) |
| **4,4’** | 24.3 | 1.81(1H, m) |
| **5,5’** | 21.3 | 0.97 (3H, d) |
| **6,6’** | 23.4 | 0.94(3H, d) |
